# Markers of Fertility in Adolescents With Chronic Endocrinopathies at Transition From Paediatric to Adult Care

**DOI:** 10.1002/edm2.493

**Published:** 2024-06-07

**Authors:** Daniela Choukair, Janna Mittnacht, Markus Bettendorf

**Affiliations:** ^1^ Division of Paediatric Endocrinology and Diabetes, Department of Paediatrics University Hospital Heidelberg Heidelberg Germany

**Keywords:** adolescents with chronic endocrinopathies, fertility markers, transition

## Abstract

**Objective:**

During the process of transition from paediatric to adult health care, counselling concerning fertility is an important issue and is based mainly on serum markers of gonadal function. Here, we analysed these markers in adolescents with various underlying endocrine diseases at the time of transition.

**Methods:**

After reaching near adult height and late puberty (girls: bone age [BA] ≥14 years, and boys: BA ≥16 years), we assessed stages of puberty according to Tanner and measured testes or ovarian volumes and serum markers of gonadal function (anti‐Mullerian hormone [AMH], inhibin B, 17β‐estradiol, testosterone).

**Results:**

One hundred and ten patients (56 females and 54 males) were included from May 2010 to March 2016 with multiple pituitary hormone deficiency (MPHD; *n* = 17), growth hormone deficiency (GHD; *n* = 35), Turner syndrome (TS; *n* = 27), short stature after being born small for gestational age (SGA; *n* = 20) and Klinefelter syndrome (KS; *n* = 11). Female and male adolescents exhibited mature secondary sexual characteristics. The levels of serum inhibin B and AMH were lower in TS and female MPHD than in GHD and SGA, each independently (*p* < 0.05). The levels of serum AMH were higher whereas serum inhibin B were lower in male MPHD and KS (*p* < 0.05). Ovary volumes were significantly smaller in patients with TS, and testicular volumes were smaller in patients with KS.

**Conclusions:**

After current established treatments with sex steroids, the development of secondary sexual characteristics was mature. However, impaired markers of fertility have been identified in patients with TS, KS and MPHD, reflecting gonadal dysgenesis in TS and KS, but gonadal immaturity in MPHD as gonadal gonadotropin stimulation is lacking throughout development. Consequently, in patients with MPHD, these markers cannot reliably predict individual fertility, which warrants consideration and incorporation in future treatment concepts.

## Graphical abstract

Pubertal development was complete after established treatments with either estradiol valerate or testosterone enantate. Markers of fertility were impaired in TS, KS and MPHD, reflecting gonadal dysgenesis in TS and KS, but immaturity in MPHD. In patients with MPHD, these markers cannot reliably predict individual fertility.

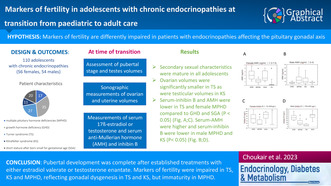

## 1 Introduction

During the transition from paediatric to adult health care, counselling regarding fertility becomes a crucial issue for adolescents with chronic endocrinopathies, particularly for those with hypogonadotropic hypogonadism (HH) as seen in hypopituitarism or hypergonadotropic hypogonadism as seen in Klinefelter syndrome (KS) or Turner syndrome (TS) [1, 2, 3]. Replacement therapy with cyclic oestrogen–progesterone in girls and with testosterone in boys aims to facilitate the maturation of secondary sexual characteristics at a physiological pace [4]. Sex steroids not only play a vital role in somatic and psychological well‐being but also impact bone mineral density, haematopoiesis and cardiovascular, sexual and metabolic health, with minimal effects on fertility [4].

Specific markers such as serum inhibin B and anti–Mullerian hormone (AMH) are utilised to predict individual fertility [2, 5, 6]. AMH, produced by granulosa cells of preantral and small antral follicles, remains relatively stable across the menstrual cycle and is increasingly recognised as a marker for ovarian reserve [7]. Inhibin B, on the contrary, indicates developing granulosa cells [2, 7] and exhibits varying serum concentrations throughout the menstrual cycle [7, 8].

Follicle‐stimulating hormone (FSH) stimulates serum AMH, which remains high from male foetal life until mid‐puberty and serves as a marker of immature Sertoli cells. In maturing testes, AMH is inhibited by intratesticular testosterone, leading to a decrease in serum levels during childhood and puberty [9]. Inhibin B also reflects Sertoli cell function and spermatogenesis, increasing during puberty and maturation of Sertoli cells. Postpuberty, inhibin B production depends on the number of Sertoli cells and correlates with sperm counts [6, 9].

The aim of this study was to evaluate markers of fertility and secondary sexual development in boys and girls at the time of transition from paediatric to adult care.

## 2 Patients and Methods

The study was prospectively designed as a single‐centre, exploratory, descriptive, open, noncontrolled study. The Ethics Committee of the University Medical Faculty Heidelberg approved the study (S‐019/2011). Written informed consent was obtained from patients and/or their respective parents prior to participation. All procedures performed were in accordance with the ethical standards of the institutional and/or national research committee, as well as the principles of the 1964 Helsinki Declaration and its subsequent amendments in 2013, or comparable ethical standards.

### 2.1 Patient Characteristics

We included a consecutive cohort of 110 adolescents (56 females and 54 males) who were followed in the Division of Paediatric Endocrinology at the Children's University Hospital Heidelberg between May 2010 and March 2016. These patients were diagnosed with various endocrine diseases including multiple pituitary hormone deficiency (*n* = 17, MPHD), growth hormone deficiency (*n* = 35, GHD), TS (*n* = 27) short stature after being born small for gestational age (*n* = 20, SGA) and KS (*n* = 11). Diagnostic and therapeutic procedures for these patients were conducted in accordance with German national or international standards, as previously reported [4, 10]. Inclusion criteria comprised (i) a chronic endocrine disease as specified above, and (ii) for girls: bone age (BA) ≥14 years and menarche, and for boys: BA ≥16 years, and pubertal voice break. Exclusion criteria were severe intellectual disability and lack of informed consent.

Two female patients with MPHD had congenital cases, whereas five had acquired MPHD. As part of the therapy, female patients with MPHD and HH received oral continuous estradiol valerate (2 mg) along with cyclic oral chlormadinone acetate 2 mg from Days 1 to 12 every month (*n* = 5). Among male patients, four had congenital MPHD, and six had acquired MPHD. Male MPHD patients with HH received testosterone either as testosterone enanthate (250 mg once per month) or testosterone undecanoate (1000 mg/once every 3 months) intramuscular (*n* = 6), whereas one patient received human chorionic gonadotropin (hCG) at doses ranging from 500 to 1500 IU subcutaneously twice a week (*n* = 1).

Sixteen out of 17 patients with MPHD received GH treatment at doses ranging from 0.025 to 0.035 mg/kg/day subcutaneously following diagnosis in childhood, which was confirmed by standard arginine and insulin tolerance tests. At the time of transition, five patients with MPHD continued to receive GH treatment after confirming GH deficiency through retesting in adolescence. Additionally, other medications included L‐thyroxine (at doses ranging from 25 to 200 μg once daily orally, *n* = 13) because of central hypothyroidism and hydrocortisone (at doses ranging from 5 to 20 mg orally, three times daily; *n* = 13) due to central adrenal insufficiency.

All patients with GHD (*n* = 35) received GH treatment at doses ranging from 0.025 to 0.035 mg/kg/day subcutaneously following diagnosis in childhood, which was confirmed by standard arginine and insulin tolerance tests. At the time of transition, two patients with GHD continued to receive GH treatment after confirming GH deficiency through retesting in adolescence.

The treatment of 21 girls with TS comprised continuous oral estradiol valerate (2 mg) along with oral cyclic chlormadinone acetate 2 mg from Days 1 to 12 every month. Among the patients with TS, six girls with X mosaicism (45 X0/46 XX [*n* = 3] and 45 X0/47 XXX [*n* = 3]) exhibited spontaneous regular menstrual cycles. Furthermore, 25 out of 27 patients with TS (92.6%) received GH treatment at doses ranging from 0.045 to 0.05 mg/kg/day subcutaneously until reaching final height, whereas two patients remained untreated as their epiphyses were already fused at the time of diagnosis.

All patients with SGA (*n* = 20) received GH treatment at a dose of 0.035 mg/kg/day subcutaneously.

Nine boys with KS received testosterone either as testosterone enanthate (250 mg once per month) or as testosterone undecanoate (1000 mg once every 3 months) intramuscularly (*n* = 6), whereas one patient received transdermal testosterone (2.5 g/daily).

### 2.2 Examinations

As part of a standardised paediatric examination conducted at the time of transition, various parameters were evaluated for adolescents who had achieved the primary paediatric treatment goals in accordance with the inclusion criteria. These parameters included chronological age (CA, years), near final height (NFH, cm), weight (kg), body mass index (BMI, kg/m^2^), serum markers of gonadal function and serum levels of sex steroid hormones. The assessment of Tanner stages and measurement of testicular volumes were performed by the same paediatrician using the Prader orchidometer [11, 12].

Additionally, volumes of left and right ovaries and uterus were determined by transabdominal ultrasound by the same radiologist. Measurements were conducted in three dimensions (length, width and height in centimetres) to accurately assess the size and dimensions of the uterus and ovaries.

### 2.3 Laboratory Analysis

AMH levels were determined using an enzyme‐linked immunosorbent (ELISA) kit (AMH Gen II; Beckman Coulter, Fullerton, CA). The lower limit of detection for AMH was 0.08 ng/mL. Inhibin B levels were measured using an ELISA assay kit (Inhibin B ELISA Kit; Ansh LABS, TX), with a lower limit of detection of 7.23 ng/L. Testosterone levels were measured via liquid chromatography‐mass spectrometry (Agilent, Santa Clara, CA, USA), whereas estradiol levels were determined using chemiluminescence immunoassay (Siemens Healthcare Diagnostic Advia Centaur XP Kit, Erlangen, Germany).

### 2.4 Statistical Analysis

Statistical analyses were conducted using SAS StatView Software, Version 5.01998. Descriptive statistics are reported as median and maximum and minimum for all variables, or frequencies, which are graphically represented in box plots indicating 10th, 25th, 50th, 75th and 90th percentiles. Group comparisons were performed using the nonparametric Kruskal–Wallis test, followed by pairwise Mann–Whitney tests with the Bonferroni correction for multiple comparisons. Correlations were assessed using the nonparametric Spearman's coefficient. Results were considered statistically significant at a threshold of *p* < 0.05.

## 3 Results

In this study, a total of 110 adolescents were included, comprising 56 females and 54 males. CA of all patients at the time of transition ranged from 14.0 to 29.6 years (median 17.5; Table 1). Patients diagnosed with MPHD were found to be significantly older, whereas those with SGA were significantly younger than other patients (*p* < 0.05). Additional details regarding NFH, BMI, serum estradiol and serum testosterone levels are presented in Table 1.

**TABLE 1:** Patient characterizations and substitution of sex steroids at the time of transition (median, minimum and maximum) grouped by individual diagnoses.

					Serum estradiol pg/mL substitution		Serum testosterone ng/mL substitution	
	Chronological age at transition	NFH (SDS)	BMI (SDS)	Substitution estradiol/testosterone	Yes	No	Yes^a^	No
GHD								
Female (*n* = 12)	16.8 (14.1–24.1)	−1.3 (−2.8 to 0.4)	0.1 (−2.2 to 1.4)			69.7 (20.1–417.7)	—	
Male (*n* = 23)	17.3 (14.8–20.2)	1.3 (−4.4 to 1.0)	0.1 (−4.2 to 2.2)	—	—		—	633.0 (236.0–1060.0)
MPHD								
Female (*n* = 7)	23.1* (15.3–29.6)	−0.6 (−2.7 to 2.4)	2.3 (0.2–3.1)	*n* = 5	84.7 (32.5–146.8)	29.1 (15.2–43.0)	—	
Male (*n* = 10)	20.7 (17.3–29.39	−0.4 (−3.9 to 0.9)	0.5 (−0.7 to 3.0)	*n* = 7	—		715.0 (241–1013)	758.0 (108–1156)
SGA								
Female (*n* = 10)	14.6* (14.0–18.3)	−1.7 (−3.6 to −0.8)	−0.6 (−1.4 to 2.5)	—	—	43.85 (24.2–348.7)	—	
Male (*n* = 10)	16.6 (15.5–17.9)	−1.9 (−2.9 to −0.8)	0.3 (−2.1 to 1.9)	—	—		—	438.0 (308–768)
TS (*n* = 27)	17.7 (14.3–24.2)	−2.7 (−3.8 to −0.9)	0.6 (−1.8 to 2.9)	*n* = 21	49.5 (0–122.6)	60.2 (17.5–283.9)	—	
KS (*n* = 11)	18.3 (15.2–20.8)	0.6 (−0.7 to 2.7)	0.3 (−2.8 to 1.4)	*n* = 10	—		522.0 (175.0–1064.0)	625.0

Abbreviations: BMI, body mass index; GHD, growth hormone deficiency; KS, Klinefelter syndrome; MPHD, multiple hormone deficiency; NFH, near final height; SDS, standard deviation score; SGA, short stature small for gestational age; TS, Turner syndrome.

^a^ One patient received human chorionic gonadotropin (500–1500 IU s.c. twice a week) and no testosterone.

* *p* < 0.05.

### 3.1 Sexual Development

#### 3.1.1 Female Patients

**FIGURE 1:**
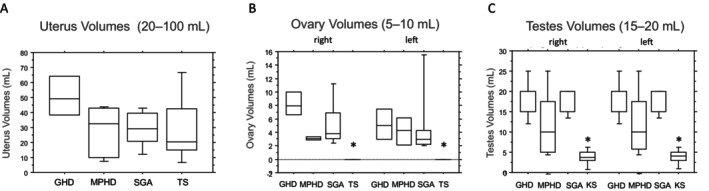
(A) Uterus volumes (mL) in female patients grouped by diagnoses. GHD (growth hormone deficiency): *n* = 4, MPHD (multiple pituitary hormone deficiency): *n* = 6, SGA (small for gestational age): *n* = 8 and TS (Turner syndrome): *n* = 27. Box plots indicate 10th, 25th, 50th, 75th and 90th percentiles. Group comparisons were made with the nonparametric Kruskal–Wallis test: Results were considered not significant at *p* > 0.05. (B) Right and left ovary volumes (mL) in female patients grouped by diagnoses. GHD: *n* = 4, MPHD: *n* = 6, SGA: *n* = 8 and TS: *n* = 27. Box plots indicate 10th, 25th, 50th, 75th and 90th percentiles. Group comparisons were made with the nonparametric Kruskal–Wallis test: Results were considered significant at *p < 0.05 compared with GHD, MPHD and SGA. (C) Right and left test volumes (mL) in male patients grouped by diagnoses. GHD: *n* = 23, MPHD: *n* = 9, SGA: *n* = 10 and KS (Klinefelter syndrome): *n* = 10. Box plots indicate 10th, 25th, 50th, 75th and 90th percentiles. Group comparisons were done with the nonparametric Kruskal–Wallis test: Results were considered significant at *p < 0.05 compared with GHD, MPHD and SGA.

In female patients, assessments of pubertal development indicated consistent findings in breast development across all individuals (median Tanner Stage 5; data not shown). Uterus volumes were measured at 32 ± 24 mL and did not demonstrate statistical differences among the patient groups (not significant; Figure 1A). However, left and right ovary volumes were found to be similar and significantly lower in patients with TS than in other patients (*p* < 0.05; Figure 1B). Serum estradiol levels did not differ significantly between girls receiving estradiol valerate substitution and those without (Table 1).

#### 3.1.2 Male Patients

Ratings of male pubertal development indicated consistent genital and pubic hair development across all male patients (median Tanner Stage 5; data not shown). However, left and right testicular volumes were found to be comparable but significantly smaller in patients with KS than in other patient groups (*p* < 0.05). Additionally, testicular volumes were slightly smaller in patients with MPHD than in those with GHD and SGA, although this difference was not statistically significant (Figure 1C). Because of the small sample size, no difference in testicular volume was detected between patients with MPHD treated with sex steroids and those left untreated. Serum testosterone concentrations were similar among all males, regardless of whether they received testosterone substitution (Table 1).

### 3.2 Markers of Fertility

#### 3.2.1 Female Patients

**FIGURE 2:**
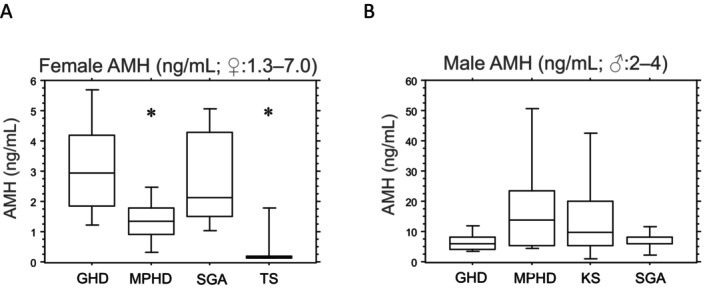
(A) Anti–Mullerian hormone (AMH: ng/mL) in female patients grouped by diagnoses. GHD (growth hormone deficiency): *n* = 14, SGA (small for gestational age): *n* = 10, MPHD (multiple pituitary hormone deficiency): *n* = 7 and TS (Turner syndrome): *n* = 27. Box plots indicate 10th, 25th, 50th, 75th and 90th percentiles. Group comparisons were made with the nonparametric Kruskal–Wallis test: Results were considered significant at *p < 0.05 compared with GHD and SGA each independently. (B) Anti–Mullerian hormone (AMH: ng/mL) in male patients grouped by diagnoses. GHD: *n* = 23, SGA: *n* = 10, MPHD: *n* = 10 and KS (Klinefelter syndrome): *n* = 11. Box plots indicate 10th, 25th, 50th, 75th and 90th percentiles. Group comparisons were made with the nonparametric Kruskal–Wallis test: Results were considered not significant at *p* > 0.05.

Patients diagnosed with TS and MPHD exhibited significantly lower serum AMH concentrations than in those with patients with GHD and SGA, each independently (*p* < 0.05; Figure 2A). Interestingly, females with MPHD demonstrated slightly higher AMH levels than those with TS.

**FIGURE 3:**
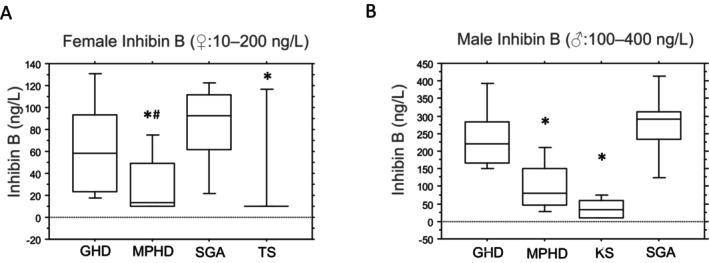
(A) Inhibin B (ng/L) in female patients grouped by diagnoses. GHD (growth hormone deficiency): *n* = 14, SGA (small for gestational age): *n* = 10, MPHD (multiple pituitary hormone deficiency): *n* = 7 and TS (Turner syndrome): *n* = 27. Box plots indicate 10th, 25th, 50th, 75th and 90th percentiles. Group comparisons were made with the nonparametric Kruskal–Wallis test: Results were considered significant at *p < 0.05 compared with GHD and SGA each independently; results were considered significant at ^#^ *p* < 0.05 compared with TS. (B) Inhibin B (ng/L) in male patients grouped by diagnoses. GHD: *n* = 23, SGA: *n* = 10, MPHD: *n* = 10 and KS (Klinefelter syndrome): *n* = 11. Box plots indicate 10th, 25th, 50th, 75th and 90th percentiles. Group comparisons were made with the nonparametric Kruskal–Wallis test: Results were considered significant at *p < 0.05 compared with GHD and SGA, each independently.

Similarly, serum inhibin B levels were significantly lower in patients with TS and those with MPHD than in patients with GHD and SGA, each independently, whereas serum inhibin B levels were slightly higher in patients with MPHD than in TS (*p* < 0.05; Figure 3A). Notably, no significant differences in inhibin B serum concentrations were observed between female patients with congenital MPHD (*n* = 2) and acquired MPHD (*n* = 5), although the sample size was limited (median 15.5 [10–21] vs. 13 [10–79]; not significant). Additionally, six girls with TS mosaicism who experienced spontaneous menarche displayed higher serum AMH and inhibin B levels than in other girls with TS (data not shown). However, no correlation was found between AMH or inhibin B levels and ovary volumes (data not shown).

#### 3.2.2 Male Patients

The level of serum AMH was higher in male patients with MPHD and KS than in those with GHD and SGA (Figure 2B). However, male patients with MPHD and KS exhibited significantly lower serum inhibin B concentrations than in those with GHD and SGA each independently (*p* < 0.05; Figure 3B), with inhibin B serum levels slightly higher in patients with MPHD than in those with KS. Male patients with congenital MPHD (*n* = 4) displayed slightly lower inhibin B levels than those with acquired MPHD (*n* = 6) (median 50 [10–115] vs. 70 [40–220]; not significant). Furthermore, a positive correlation was observed between testicular volumes and serum inhibin B concentrations in all male patients (*r*_s_ = 0.661 left testes and *r*_s_ = 0.665 right testes; *p* < 0.05). However, no correlations were detected between testicular volumes and AMH.

## 4 Discussion

This study represents the first comprehensive evaluation of serum markers of fertility and secondary sexual characteristics in adolescents with chronic endocrine diseases during the transition period. Our findings highlight the efficacy of current treatments for hypogonadism with sex steroids in facilitating the development of adequate secondary sexual characteristics. In female patients, lower serum levels of AMH and inhibin B were observed in those with TS and MPHD than in patients with GHD and SGA each independently. This discrepancy in AMH and inhibin B levels may reflect dysfunction of granulosa cells, follicular arrest and low oocyte number in patients with TS [13, 14]. In these patients, premature and accelerated follicle atresia can manifest during foetal life, childhood or early adulthood leading to the development of small or streak gonads [13]. In our cohort, six girls with a mosaic karyotype (22.2%) experienced spontaneous puberty, a proportion comparable to the 10% reported by Karnis [14]. However, Borgstrom et al. reported that many girls with TS have follicles present in their ovaries during adolescence, even in the absence of spontaneous puberty or with high serum concentrations of LH and FSH, along with low AMH levels [15]. Notably, cryopreservation of primordial follicles within ovarian cortical tissue should be considered for all patients with TS and their families [16].

Similarly, female patients with MPHD displayed low serum inhibin B and AMH levels, potentially attributed to gonadotropin deficiencies limiting follicular maturation [17, 18]. This hypothesis is supported by in vitro findings indicating that postnatal ovarian development in hypogonadal female mice lacking gonadotropins is devoid of antral follicles [19]. Furthermore, research has demonstrated the significance of FSH in the development of the very early follicular stages of human ovarian tissue in vitro [20]. However, caution is warranted in using AMH as a diagnostic marker for estimating ovarian reserve in female patients with prepubertal onset of HH, as it may not accurately reflect the ovarian reserve because of gonadotropin deficiencies [7].

In male patients, slightly lower inhibin B levels were observed in those with congenital MPHD and HH than in acquired MPHD, likely due to fewer Sertoli cells and reduced germ cell count resulting in smaller testes [21, 22]. Serum inhibin B levels demonstrated a positive correlation with testicular volumes [21], indicating its utility as a marker for discriminating HH from constitutional delay of puberty [23]. Additionally, males with KS exhibited lower inhibin B levels but higher AMH levels, reflecting Sertoli cell dysfunction with impaired spermatogenesis. Low inhibin B causes impaired feedback on FSH resulting in higher serum FSH levels [24]. In patients with KS, Leydig cell exhaustion variably occurs during late puberty [25].

Despite exogenous testosterone treatment in male patients with MPHD and HH or KS, secondary sex characteristics were induced without affecting spermatogenesis or down‐regulating AMH levels [26, 27]. Thus, fertility issues should be discussed prior to testosterone treatment initiation, with consideration given to surgical procedures for testicular sperm extraction (TESE or micro‐TESE) and cryoconservation for future assisted reproductive techniques [28, 29].

Counselling concerning fertility should be integrated into transition programs for patients with chronic endocrine diseases, particularly in those with dysgenetic gonads such as TS and KS [4, 30]. Gonadotropin treatment should be discussed as an option for patients with MPHD and HH, along with FSH priming before hCG therapy to enhance testicular growth. Long‐term studies are needed to assess their impact on adult fertility [31]. In patients with congenitally acquired MPHD and HH, detecting low serum fertility markers may represent a diagnostic pitfall and cannot reliably predict individual fertility, which warrants consideration and incorporation in future treatment concepts. Although this study provides valuable insights, its single‐centre design and small sample sizes across patient subgroups represent limitations, warranting further investigation in larger, multicentre studies.

## 5 Conclusion

The current established treatments with sex steroids induce complete mature development of secondary sexual characteristics. However, notable deficiencies in fertility markers have been delineated among patients diagnosed with TS, KS and MPHD. In contrast to patients with TS and KS characterised by dysgenetic gonads, individuals with MPHD present with immature gonads displaying impaired levels of AMH and inhibin B, attributed to the absence of gonadal gonadotropin stimulation. Consequently, in patients with MPHD, these markers cannot reliably predict individual fertility, which warrants consideration and incorporation in future treatment concepts.

## Author Contributions

**Daniela Choukair:** conceptualization (equal), data curation (equal), formal analysis (equal), supervision (equal), validation (equal), writing–original draft (lead). **Janna Mittnacht:** conceptualization (equal), data curation (equal), formal analysis (equal), funding acquisition (lead), methodology (equal), project administration (equal), resources (equal), software (equal), validation (equal). **Markus Bettendorf:** conceptualization (equal), data curation (equal), formal analysis (equal), funding acquisition (equal), investigation (equal), methodology (equal), project administration (equal), supervision (equal), writing–original draft (equal).

## Ethics Statement

The study was approved by the Ethics Committee of the University of Heidelberg (S‐019/2011) and was performed in accordance with the current version of the Declaration of Helsinki.

## Consent

Written informed consent was obtained from patients and their legal guardians for participating in and publication of this study.

## Conflicts of Interest

The authors declare no conflicts of interest.

## Data Availability

The data that support the findings of this study are available from the corresponding author upon reasonable request.
